# Randomized controlled trial of all-inside and standard single-bundle anterior cruciate ligament reconstruction with functional, MRI-based graft maturity and patient-reported outcome measures

**DOI:** 10.1186/s12891-022-05231-x

**Published:** 2022-03-26

**Authors:** Rubing Lin, Qiuwen Zhong, Xiao Wu, Lei Cui, Rong Huang, Qianhua Deng, Jianwei Zuo, Changqing Jiang, Wei Li

**Affiliations:** 1 Peking University Shenzhen Hospital, Clinical College of Anhui Medical University, Lianhua Road 1120, Futian District, Shenzhen City, Guangdong Province 518036 People’s Republic of China; 2 Peking University Shenzhen Hospital, Lianhua Road 1120, FuTian District, ShenZhen City, GuangDong Province 518036 People’s Republic of China; 3 Huazhong University of Science and Technology Union Shenzhen Hospital, Taoyuan Road 89, Nanshan District, Shenzhen City, GuangDong Province 518036 People’s Republic of China

**Keywords:** All-inside, Anterior cruciate ligament reconstruction, Single-bundle, Graft maturity, Knee laxity

## Abstract

**Background:**

All-inside anterior cruciate ligament reconstruction (ACLR) is a novel technique that has gained attention due to its minimally invasive and graft-saving properties. However, studies comparing MRI-based graft maturity between all-inside and standard ACLR are lacking.

**Purpose:**

This study focused on the functional, knee laxity, and MRI-based graft maturity characteristics of all-inside and standard single-bundle ACLR.

**Study Design:**

Randomized controlled trial (RCT).

**Methods:**

Fifty-four patients were randomly assigned to an all-inside reconstruction group (*n* = 27) or standard reconstruction group (*n* = 27). Using the same rehabilitation strategy. The Tegner, International Knee Documentation Committee, and Lysholm scores were recorded at postoperative months 3, 6, and 12 to assess functional recovery. MRI was conducted to measure the signal/noise quotient (SNQ) of the intra-articular graft to assess the maturity. A higher SNQ indicates lower graft maturity. Knee laxity was assessed using GNRB arthrometer at the postoperative month 12.

**Results:**

The graft SNQ of the all-inside group was significantly higher than that of the standard group at postoperative month 6 (*p* < 0.05). There was no statistical difference in graft SNQ between the two groups at postoperative months 3 and 12 (*p* > 0.05). Both groups exhibited the highest SNQ in the middle region of the graft, followed by the proximal region, and the distal region. Functional scores improved significantly for both groups and had no statistical difference (*p* > 0.05). The knee laxity was higher in the all-inside group (*p* < 0.05) at postoperative month 12. There was no correlation between the functional scores and graft maturity in both groups (*p* > 0.05).

**Conclusions:**

All-inside and standard single-bundle ACLR show good functional outcomes; however, knee laxity was relatively higher in the all-inside ACLR group than in the standard ACLR group. Moreover, both techniques exhibited poor maturity in the middle graft region and the best in the distal region. Graft maturity with all-inside ACLR is inferior to that with standard ACLR in the early postoperative stages. There is no correlation between knee function and graft maturity.

**Trial registration:**

Clinical trial registration numbers: ChiCTR1800018543.

Date of registration: 09/23/2018.

## Introduction

Anterior cruciate ligament (ACL) tear is one of the most common sports-related injuries [1]. The odds of developing knee osteoarthritis after ACL injury are approximately four times higher than in a non-injured knee [2]. Mayo and Robson [3] first attempted to repair ACL in 1895. Subsequently, in 1995, the standard anatomic anterior cruciate ligament reconstruction (ACLR) gradually became mainstream and continues to be so to this day. In pursuit of a more minimally invasive technique, Lubowitz [4] proposed the first generation of the all-inside ACLR technique based on its predecessors in 2006 and its modified versions in 2011 [5]. The primary technical difference between our standard and all-inside ACLR technique is establishing the tibial tunnel and graft fixation. The former uses a full tibial tunnel and screw compression fixation, whereas the latter uses a bone socket structure and cortical suspension fixation.

Many studies have concluded that there is no difference between all-inside and standard ACLR in terms of functional recovery and that all-inside ACLR is better than the latter in terms of pain reduction and slowing tunnel widening [6–10]. Lubowitz thought that the bone socket structure used in all-inside ACLR could reduce the accumulation of bioinvasive factors that slows down the widening of the tunnel [11]. However, some authors have concluded that knee stability after all-inside ACLR is insufficient [12] and the graft failure rate is higher than that after standard ACLR [13]. The reason for the inadequate stability and high refurbishment rate after all-inside ACLR remains highly controversial. We hypothesize that this may be related to the premature return to motion when the graft is immature or at least not fully healed. However, studies investigating MRI-based graft maturity of all-inside and standard ACLR are lacking. Therefore, this study focused on the functional, knee laxity, and MRI-based graft maturity characteristics of the all-inside and standard single-bundle ACLR techniques 1 year postoperatively to better assess the differences in outcomes.

## Methods

This was a prospective randomized controlled trial and was approved by the hospital’s ethics review committee and registered at the national clinical registration center.

### Participants

Patients who underwent single-bundle ACLR in our department between September 2018 and July 2019 and meet the criteria were included in the study. All patients were randomly assigned to the all-inside ACLR group or the standard ACLR group using the random number table method. The sample size was determined via power analysis. The inclusion criteria for participants were history of trauma and MRI showing ACL discontinuity or hyperintense signal, age 18–45 years, body mass index (BMI) of 18–28, and informed consent and signed relevant documents. The patients were excluded if they had any of the following: injured > 12 months ago; combined collateral ligament, posterior cruciate ligament, cartilage injury of more than 3 degrees and a meniscus grade III injury; history of ACLR surgery; generalized ligament laxity; severe underlying diseases or uncooperative follow-up; and bilateral ACL injury.

### Clinical evaluation

**Fig. 1:**
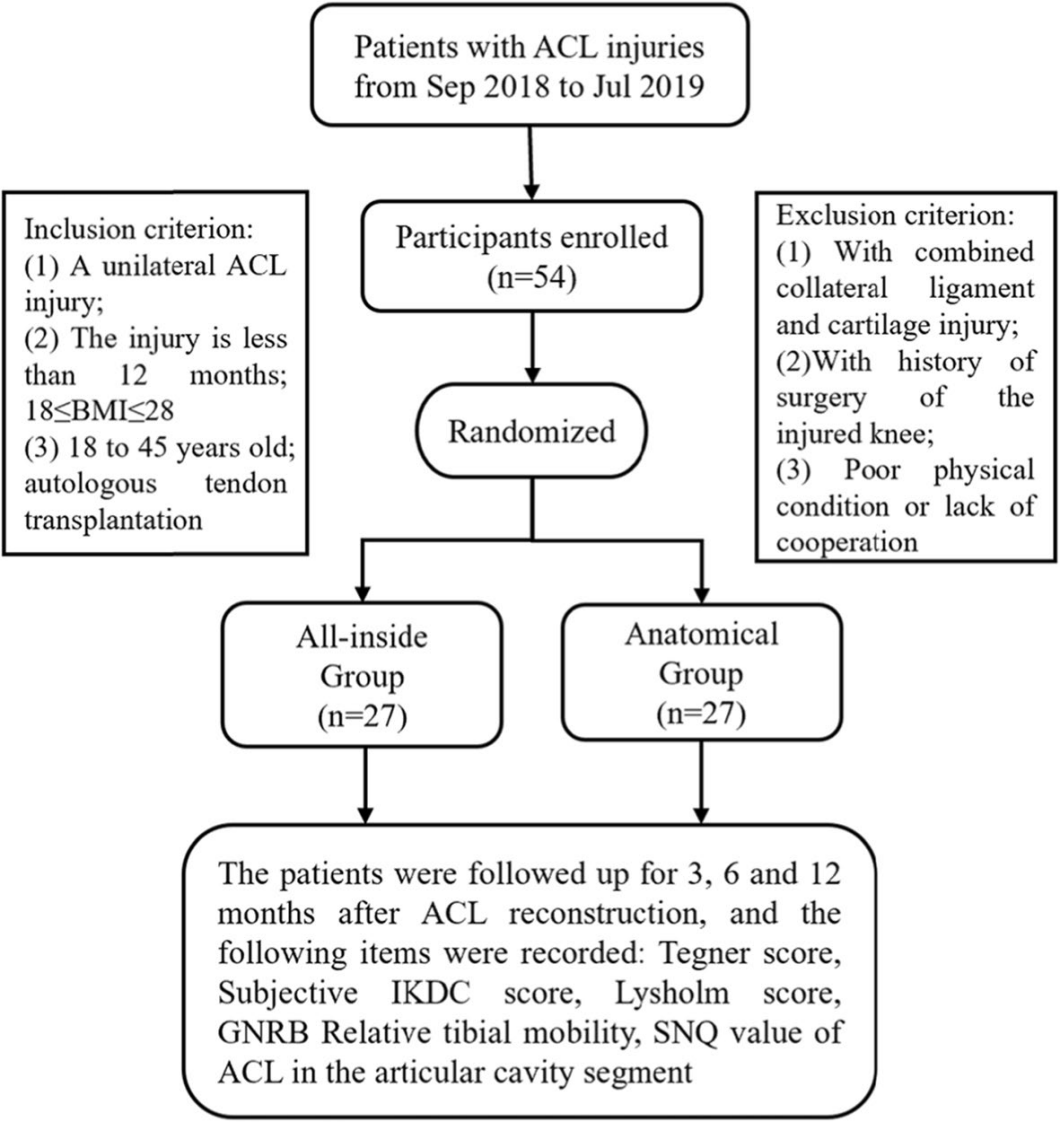
Flow diagram of the whole study

The Tegner, International Knee Documentation Committee (IKDC), and Lysholm scores were recorded at postoperative months 3, 6, and 12 to assess functional recovery. At postoperative month 12, a GNRB arthrometer (Genourob, France) was used to assess knee laxity. Arthrometer measurement was performed as follows, under knee flexion of 30°and at 134-N load, the tibia anterior movement of the ACLR and normal contralateral knee was measured separately. The difference is obtained by subtracting the two measurements. The measurement was repeated three times and the value with the greatest difference was taken. The greater the anterior laxity, the more relaxed the knee joint. Data collectors and patients were not aware of the surgical approach. Only the surgeon was aware of the grouping. The study procedure is indicated in Fig. 1.

### MRI analysis

The signal/noise quotient (SNQ) of the proximal, middle and distal regions of interest (ROI) of the intra-articular graft was measured using 3.0-T MRI (Extremity 18 3 T Tim Coil, SIEMENS, Germany) at postoperative months 3, 6, and 12 to observe the graft maturity law. All patients were required to rest for 60 min before measurement. Using fat-suppressed proton density (FS-PD) sequence. Thickness was 3 mm. Repetition time (TR) was 3140 ms. Echo time (TE) was 36 ms. Flip angle was 150°. Scan time was 118 s. Matrix was 320 × 256. Field of view (Fov) was 160 mm. From a sagittal image (Pd-tse-fs-sag) to select the interface that clearly showed graft and quadriceps tendon ROI. The graft ROI was located at the proximal (near the femoral tunnel), distal (near the tibial tunnel), and the middle (between them) regions. The ROI for the quadriceps tendon was located 2 cm above the patellar attachment point [14]. The signal value of the point 2 cm anterior to the tibial tuberosity was used as the background signal. The area of ROI signal measurement was controlled at 10-sq.mm (with an allowable error of ≤ 0.5-sq.mm). The MRI-based graft SNQ is used widly to assess graft maturity [15]. A higher SNQ indicates lower graft maturity. Graft SNQ was calculated using the following formula.

Graft SNQ = (Graft ROI mean signal value − Quadriceps tendon ROI mean signal value) / Background mean signal value.

**Fig. 2:**
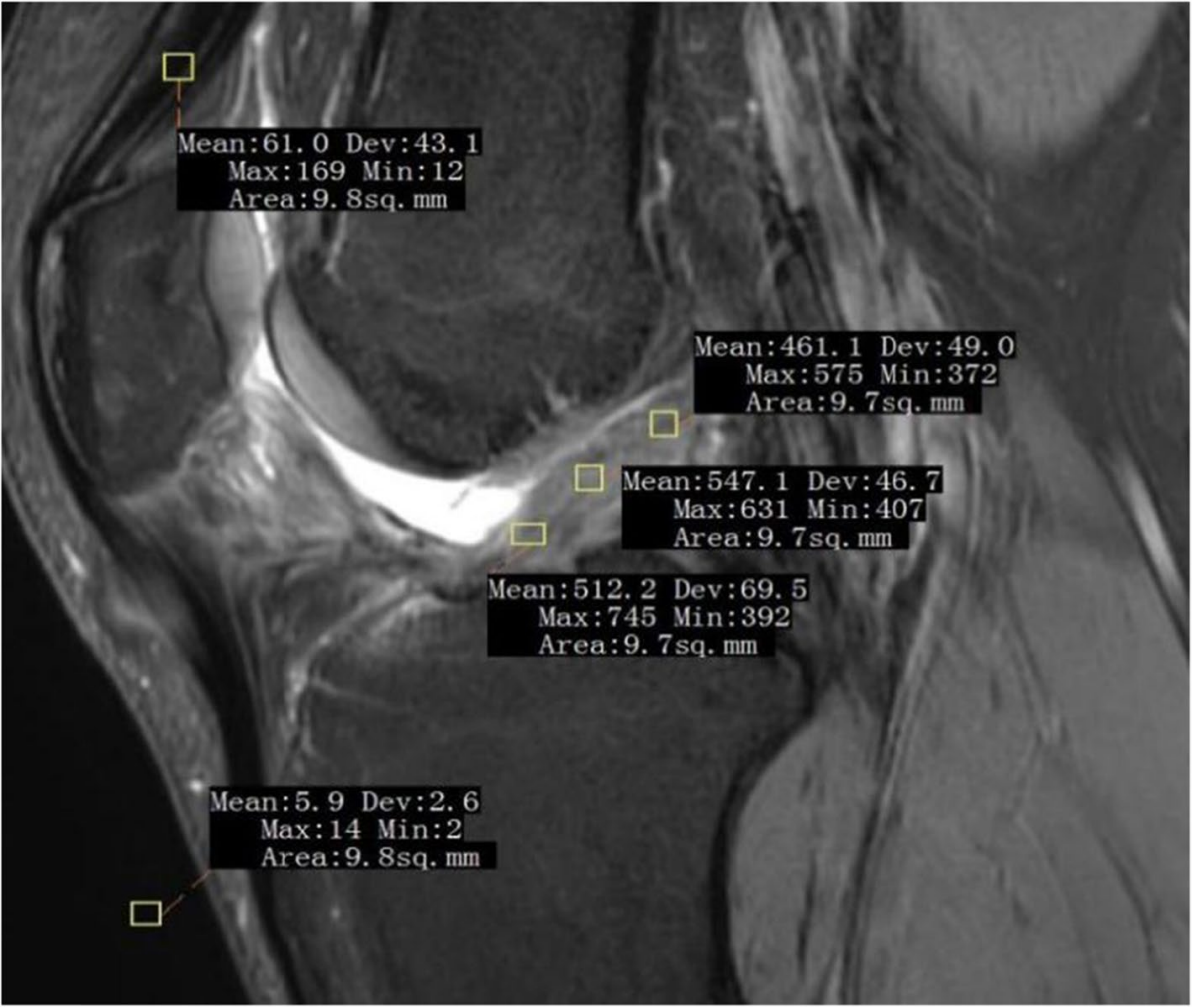
Location of the proximal, distal, and middle regions of interest (ROI) of the intra-articular graft, and the location of the quadriceps tendon and background ROI

The MRI scan was perform at a single magnet for all patients, and the whole process was completed by a fixed, blinded radiologist. The location of the proximal, distal, and middle ROI have been presented in Fig. 2.

### Surgical techniques

All operations were performed by Prof. W Li who is an experienced orthopedic trauma surgeon specializing in sports medicine. The patient was placed in the supine position after lumbar anesthesia, with the lower leg hanging over the edge of the bed. A subpatellar anteromedial and anterolateral approach was used to perform knee arthroscopy, and a planing knife was used to clean the subpatellar fat pad and synovial membrane. The injury was explored, the ACL tibial and femoral stumps were cleaned, and the posterior edge of the lateral wall of the intercondylar socket of the femur was exposed. Using an anteromedial approach, the entry point was prepped using a microfracture cone and marked slightly posteriorly above the midpoint of the lateral wall of the resident’s crest.

**Fig. 3:**
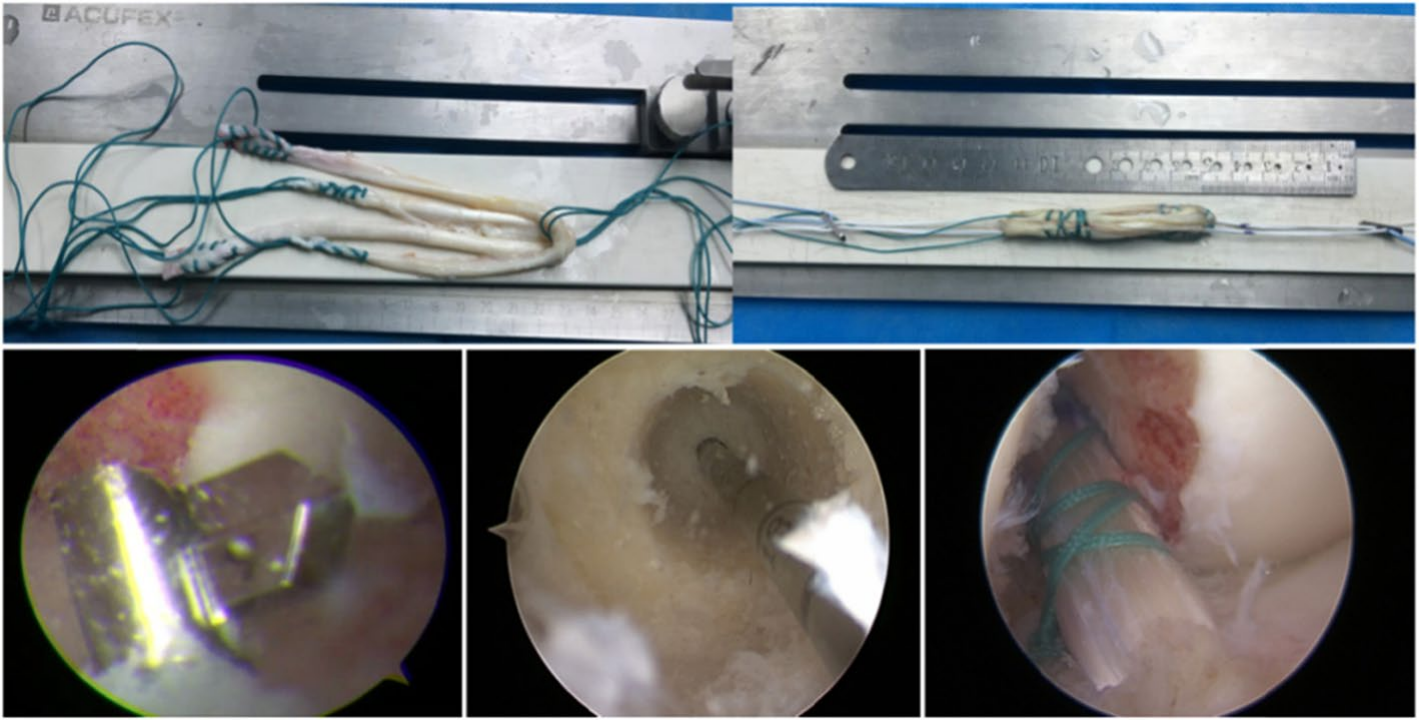
Tendon graft preparation and drilling of the tunnel bone socket structure in the all-inside single-bundle anterior cruciate ligament reconstruction technique

The all-inside reconstruction technique was performed via an anteromedial approach using an inverted drill (Arthrex, USA) to drill a 2-cm hole in the femoral bone tract in a retrograde fashion (Fig. 3). Standard reconstruction was also performed via an anteromedial approach using an ordinary drill to drill the bone tract in a prograde fashion, with the size of the bone tract depending on the diameter of the grafted tendon. For creating the tibial tunnel, both reconstruction techniques used an internal port that was located at the center of the C-shaped stop of the ACL tibia and an external port located approximately 3 cm medially to the tibial tuberosity. For the all-inside reconstruction technique, the tibial tunnel was drilled using an inverted drill (Arthrex, USA), and the drill wing was opened to enlarge the tract in a retrograde fashion to a depth of approximately 2 cm. For the standard reconstruction technique, a guide pin was first placed and the full-length tibial tunnel was drilled along the guide pin.

The autologous tendons were collected for graft preparation before drilling the tunnel. The size of the graft was used to determine the size of the tunnel. Both techniques were performed by making an oblique incision on the skin at approximately 3 cm medial to the tibial tuberosity to expose the semitendinosus and gracilis tendon, and the tendon was retrieved using a tendon retriever. All-inside reconstruction involved only a single harvest of the semitendinosus tendon, and the extracted tendon was double-folded to create a four-stranded single-bundle structure (Fig. 3) The ends of the graft tendon were connected with an adjustable TightRope (Arthrex, USA). The diameter and length of the graft were recorded intraoperatively. The prepared grafted tendon was entered through the arthroscopic port, and the retraction cord guided the grafted tendon through the femoral tunnel. The adjustable steel plate was then attached outside the bone cortex and fixed by tightening and knotting it. Next, the tibial guide cord was tractored to the tibial tunnel along with the lead on the other side of the grafted tendon, and the traction tibial guide cord pulled the tendon lead out, pulling the tendon into the tibial tunnel. Standard reconstruction involved harvesting the semitendinosus and gracilis tendon, folding them in half to prepare a four-stranded single-bundle structure, and attaching an adjustable steel plate to one end of the graft tendon, and securing it with an ordinary traction wire at the other end. The graft end with an adjustable steel plate was guided through the tibial tunnel to the femoral tunnel, and the steel plate was then stuck outside the femoral cortex and fixed by knotting it. Another graft end on the tibial side was fixed with an absorbable interference screw (DePuy Mitek, USA).

For standard ACLR, the graft was tightened using the traction rope after placement, and the knee was stretched and flexed 10 times. Finally, the knee was tightened at 15 degrees of flexion and fixed with an interference screw. For all-inside ACLR, only the adjustable loop was tightened and knotted outside the tibial tunnel when the knee was flexed at 15 degrees. After the graft was completely fixed, it was confirmed via arthroscopy that the tendon provided no torsion, the tension was good, and there was no impact between the graft and the femoral condyle. The joint cavity was flushed, and the incisions were sutured and bandaged, and the affected limb was fixed with a straight leg brace. The patient was then returned to the ward.

### Postoperative rehabilitation

The rehabilitation protocol for both groups was the same. The patient was gradually weight-bearing on the affected limb from the third day to the first week postoperatively. From weeks 2 to 4, the patient practiced knee flexion with a goal of 90 degrees. During postoperative week 4, the use of crutches was stopped, and full weight-bearing was achieved. During postoperative month 3, the knee was flexed to 130 degrees, and squatting exercises against the wall were started. Flexibility and technique training was started in postoperative month 4, and patients returned to \to non-contact sports activities at postoperative month 6 and to pivoting sports including competitive, confrontational, physical contact sports at postoperative month 9.

### Statistical analyses

Power analysis was used to determine the sample size (α err prob = 5%, 1-β err prob = 80%). IBM SPSS 21.0 statistical software was applied to outcomes data analysis. The measurement data were expressed as mean ± standard deviation (M ± SD). For comparison between two independent groups, the data met the normal distribution criteria using independent samples t-test and Pearson correlation analysis. The data did not meet the normal distribution using Mann–Whitney U-test and Spearman correlation analysis. A *p* value of < 0.05 was considered statistically significant.

## Results

### Patient demographics

Fifty-four patients who underwent ACLR at our department were randomly assigned to the all-inside group (*n* = 27) or the standard group (*n* = 27). During the follow-up, one patient in the standard group and two in the all-inside group were lost follow-up as they lived abroad. At the end of the follow-up period, the all-inside and standard groups included 25 and 26 patients, respectively. There were no statistical differences in the demographic data of the two groups (*p* > 0.05). The graft length was significantly shorter in the all-inside group than in the standard group (*p* < 0.001). There was no statistically significant difference in the graft diameters of the two groups (*p* > 0.05; Table 1).

**Table 1:** Demographic data and graft size of the participants

Demographic Data	All-inside group (*n* = 25)	Standard group (*n* = 26)	*P* value
Men:women, n	22:3	23:3	n.s
Operative side, left/right, n	9/16	12/14	n.s
Age, mean ± SD, y	31.3 ± 5.8	29.9 ± 4.6	n.s
Body mass index, mean ± SD, kg/m^2^	24.0 ± 3.2	23.6 ± 2.5	n.s
Diameter of grafts, mean ± SD, mm	7.9 ± 0.3	7.8 ± 0.3	n.s
Length of grafts, mean ± SD, cm	6.6 ± 0.2	8.1 ± 0.3	< 0.001^a^

n.s., no significant difference between the two groups, *p* > 0.05

^a^Statistical significance between the two groups, *p* < 0.05

### Clinical findings

There were no statistical differences in the Lysholm, Tegner, and IKDC scores of the all-inside and standard group (*p* > 0.05) preoperatively and at postoperative months 3, 6, and 12. Knee stability was restored in both groups at postoperative month 12, but arthrometery showed that the difference in the tibial anterior movement of the ACLR and normal contralateral knees was significantly greater in the all-inside group than in the standard group (*p* = 0.048; Table 2).

**Table 2:** Clinical outcomes in the all-inside and standard groups

Groups	Preoperative^a^	3 months^a^	6 months^a^	12 months^a^
**All-inside group**				
Tegner score	2.70 ± 1.84	2.70 ± 0.95	4.15 ± 1.13	6.70 ± 0.95
IKDC score	50.98 ± 22.03	60.88 ± 14.52	73.96 ± 11.95	87.37 ± 6.72
Lysholm score	58.35 ± 22.18	76.52 ± 14.40	86.41 ± 9.41	94.19 ± 6.16
GNRB	N	N	N	**2.87 ± 1.23**^b^
**Standard group**				
Tegner score	2.78 ± 1.85	2.59 ± 0.84	4.44 ± 1.01	6.52 ± 1.05
IKDC score	47.04 ± 22.53	59.74 ± 13.82	78.34 ± 9.20	86.86 ± 8.18
Lysholm score	55.00 ± 22.63	76.74 ± 12.53	87.81 ± 6.93	92.67 ± 5.76
GNRB	N	N	N	**2.27 ± 1.34**^b^

^a^Clinical outcomes in the all-inside and standard groups before operation and then at postoperative months 3, 6, and 12

Values are shown as mean ± SD; N, not measured

^b^Comparison between the two groups show a significant difference, *p* < 0.05

### Graft maturity on MRI

**Fig. 4:**
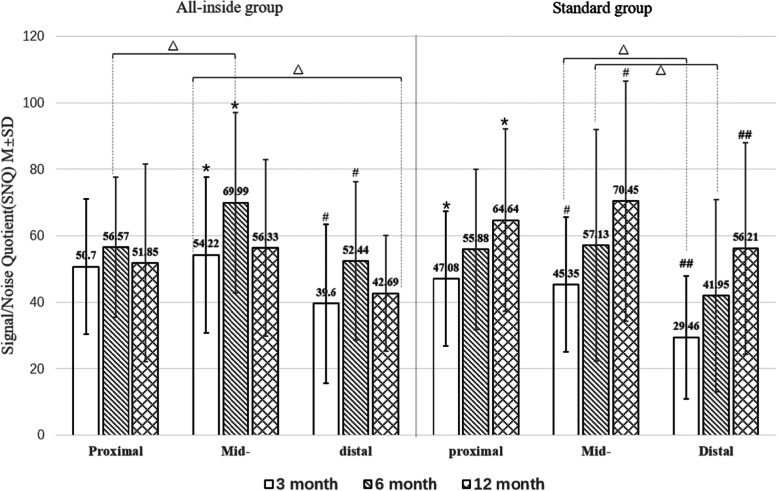
Signal/noise quotient (SNQ) of the proximal, distal, and middle ROIs of the all-inside and standard groups at postoperative months 3, 6, and 12. Both groups showed the highest SNQ in the middle region of the graft, followed by the proximal region, and the distal region. The SNQ of the all-inside group was the highest in June and then decreased, whereas the SNQ of the standard group gradually increased. #, ##, _*,_ and △ indicate a statistical difference. M ± SD, mean ± standard deviation

There were no cases of revision surgery in either group, and there were no adverse effects such as infection and instability or stiffness of joints. MRI was performed at postoperative months 3, 6, and 12. The graft SNQ in the all-inside group tended to increase from postoperative month 3 to month 6 and gradually decreased from postoperative month 6 to month 12. The graft SNQ in the standard group gradually increased with time during the follow-up period. Both groups showed the highest SNQ in the middle ROI of the graft, followed by the proximal region, and the lowest was the distal region (Fig. 4).

**Fig. 5:**
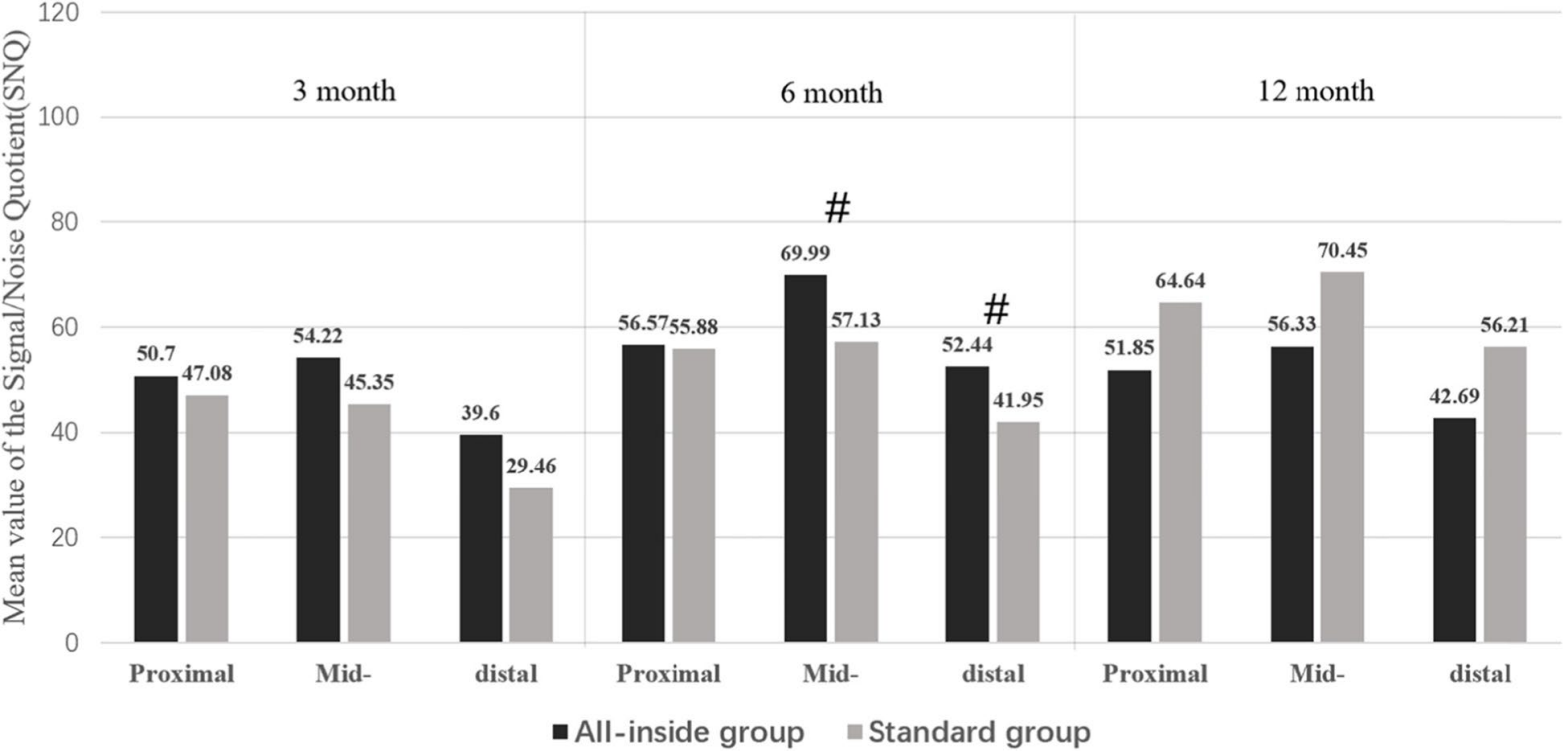
Signal/noise quotient (SNQ) of the proximal, distal, and middle ROIs of the all-inside and standard groups at postoperative months 3, 6, and 12. The SNQ of the all-inside group in the middle and distal regions was significantly higher than that of the standard group at postoperative month 6 (*p* < 0.05). There was no statistical difference between the rest of the time or region (*p* > 0.05)

There was no statistical difference in the graft SNQ between the two groups at postoperative months 3 and 12 (*p* > 0.05). The graft SNQ increased in both groups in postoperative month 6 than at postoperative month 3. Nonetheless, the increase in the all-inside group was more obvious and reached a peak; the difference between the two groups was significant (*p* < 0.05; Fig. 5).

### Correlation analysis

Correlation analysis was conducted for the functional scores and SNQ of the proximal, distal, and middle ROIs of the all-inside and standard groups at postoperative months 3, 6, and 12. There was no correlation between the functional scores and graft maturity for both groups (*p* > 0.05; Table 3).

**Table 3:** Correlation analysis between functional scores and graft SNQ^b^

*p* value/correlation coefficients^a^		All-inside group (*n* = 25)			Standard group (*n* = 26)		
		**Proximal**	**Middle**	**Distal**	**Proximal**	**Middle**	**Distal**
**Tegner**	**3 m**	**0.67 / 0.09**	**0.64 / 0.10**	**0.52 / 0.13**	**0.43 / 0.16**	**0.47 / 0.14**	**0.98 / 0.01**
	6 m	0.68 / 0.08	0.74 / 0.07	0.35 / 0.19	0.24 / -0.23	0.93 / -0.02	0.83 / 0.04
	12 m	0.22 / -0.25	0.24 / -0.23	0.34 / -0.20	0.98 / -0.01	0.93 / 0.02	0.91 / 0.02
**IKDC**	**3 m**	**0.46 / -0.15**	**0.94 / 0.02**	**0.54 / 0.12**	**0.10 / 0.32**	**0.15 / 0.28**	**0.99/ 0.004**
	6 m	0.73 / -0.07	0.66 / -0.09	0.82 / -0.05	0.67 / 0.09	0.11 / 0.32	0.80 / 0.05
	12 m	0.81 / -0.05	0.42 / -0.16	0.58 / -0.11	0.40 / 0.17	0.42 / 0.16	0.97 / 0.01
**Lysholm**	**3 m**	**0.86 / 0.04**	**0.36 / 0.18**	**0.70 / 0.08**	**0.17 / 0.27**	**0.27 / 0.22**	**0.80 / 0.05**
	6 m	0.95 / 0.01	0.63 / -0.10	0.93 / 0.02	0.19 / 0.26	0.67 / 0.09	0.74 / -0.07
	12 m	0.40 / -0.17	0.16 / -0.28	0.81 / -0.05	0.10 / 0.33	0.16 / 0.28	0.06 / 0.36

^**b**^Correlation analysis between functional scores and the SNQ of the proximal, distal, and middle ROI of the all- inside and standard group at postoperative months 3, 6, and 12

^**a**^All *p* values were > 0.05; there was no significant correlation

## Discussion

Intra-articular biohealing of grafts after ACLR includes a cell necrosis phase within 3 months postoperatively, cell regeneration revascularization and graft remodeling phase after three months [16–18]. This study found no significant differences in the graft maturity of the all-inside and standard ACLR groups at 3 months postoperatively. This suggests that both techniques follow a similar healing process during hematologic reconstitution stages. However, the graft maturity after all-inside ACLR was significantly lower than that after standard ACLR 6 months postoperatively. This may be related to chronic mechanical injuries, including “bungee” and “wiper” caused by suspension fixation when the frequency of exercises increases [19]. In contrast, standard ACLR interference screw fixation can provide a stable environment before surrounding bone resorption. However, graft maturity after standard ACLR at 1 year postoperatively was not better than that after all-inside ACLR. Putnis et al. [20] came to the same result that all-inside and standard ACLR provided comparable MRI graft signal intensity at 1 year postoperatively. This reversal might have occurred due to tunnel widening. Zhang et al. revealed that tunnel widening was associated with poor graft maturity [21]. Most studies have shown that tunnel widening is more significant after standard ACLR than after all-inside ACLR [10, 20]. The reason may be that interfering screw increases the diameter of the tunnel and promotes bone resorption, resulting in continuous erosion of synovial fluid. Because bioerosion factors also play an important role in tunnel widening [22].

The results also revealed that the standard ACLR graft SNQ gradually increased during 1-year follow-up. Nonetheless, Van Dyck et al*.* [23] concluded by systematic evaluation that the graft SNQ of standard ACLR peaked 6 months postoperatively and decreased over time. The two results are inconsistent; however, the differences may be related to the different rehabilitation strategies and the need for the study population to return to exercise and work postoperatively. Patients with aggressive rehabilitation strategies or high postoperative exercise as well as the need to return to work quickly may have poor compliance and overactivity, leading to delayed graft maturation postoperatively. In addition, there is evidence of continuous metabolic activities in the graft within 1 year, and it can last up to 2 or more years [21, 24, 25]. There may be significant individual variability in the biological healing of the graft. Therefore, any premature or excessive motor state before completing graft ligamentization may delay graft maturity; this may have contributed to the delayed appearance of the SNQ-decreasing trend in standard ACLR in our study.

According to our study, all-inside and standard ACLR exhibited the worst maturity in the middle graft region, followed by the graft near the femoral tunnel; the tibial side showed the best maturity. Putnis et al. [26] determined that the graft signal near the tibial tunnel was significantly lower than that of the femoral side. As the middle region of the graft is the primary area subjected to distraction forces and is far from the tunnel, graft hematologic reconstruction may be slower than the two ends. Some studies have shown that ligament stump preservation around the tunnel hole positively promotes graft maturation [27]. This suggests that graft healing may first occur in the bone tunnel and the two ends before progressing to the middle region of the graft. Therefore, the middle graft region is at a disadvantage and subjected to more distraction injury, contributing to its worst maturity. The graft near the femoral tunnel is less mature than that at the tibial side, probably because the angle between the graft and the femoral tunnel hole is large. In contrast, the tibial side of the graft is more vertical; some studies have shown that the graft-bending angle can affect early maturity [28]. Additionally, the femoral side graft passes between the femoral condyles. Therefore, there is a risk of impingement during flexion and extension activities, thus delaying the maturity of the femoral side graft.

Notably, knee laxity after all-inside ACLR is not better than that after standard ACLR, which may be related to returning to exercise prematurely after all-inside ACLR. Darren et al. [29] showed that 69.2% of studies on all-inside techniques allowed cutting and rotational movements at 6–9 months postoperatively. It may be that most patients have good functional improvement at 6–9 months postoperatively. However, our research shown that the graft maturity of all-inside ACLR was significantly worse than that of the standard ACLR at 6 months postoperatively, and there is no correlation between functional recovery and graft maturity, which is in agreement with the studies of Marcus and Zhang et al. [14, 30]. Therefore, using a more conservative rehabilitation strategy, all-inside ACLR is expected to have better performent than standard ACLR in improvving knee laxity, althoug further evidence is still needed.

### Limitations

This study had several limitations. First, the small sample size resulted in low statistical power; therefore, a study with larger sample size is required to draw more reliable conclusions. Second, data on clinical outcomes and SNQ follow-up time were limited, making the exact change tendency at 2–5 years unclear. Third, the SNQ based on MRI mostly reflected the water content in the graft, which is an indirect evidence of graft maturity. Finally, there was no standard method of measuring the SNQ of ACLR graft; therefore, we chose the quadriceps tendon for tissue normalization; however, this does not rule out deviations in measurement technology.

## Conclusions

All-inside and standard single-bundle ACLR show good functional outcomes; however, knee laxity was relatively higher after all-inside ACLR. In addition, both techniques exhibit the worst maturity in the middle graft region and the best in the distal region. All-inside ACLR graft maturity is inferior to standard ACLR at the early postoperative stages as it requires a more conservative rehabilitation strategy. There is no correlation between knee function and graft maturity.

## Data Availability

The datasets used and/or analyzed in the current study are available from the corresponding author on reasonable request.
